# Silver Diamine Fluoride in Oral Health Policy: A Strategic Pathway to Improve Access and Equity

**DOI:** 10.1111/jphd.70066

**Published:** 2026-07-23

**Authors:** Aisan Nouri, Lisa Jamieson, Seyed Kian Haji Seyed Javadi

**Affiliations:** ^1^ Australian Research Centre for Population Oral Health (ARCPOH), Faculty of Health and Medical Sciences Adelaide University Adelaide Australia

**Keywords:** community dentistry, dental caries, health equity, oral health policy, public health dentistry, silver diamine fluoride

## Abstract

**Objective:**

To examine the potential role of silver diamine fluoride (SDF) as a public health intervention and propose a practical policy framework for its equitable integration into oral healthcare systems.

**Methods:**

A policy‐oriented narrative synthesis of current evidence was undertaken, drawing on systematic reviews, clinical trials, economic evaluations, professional guidance, and implementation literature. Evidence was considered across clinical, workforce, economic, regulatory, and equity domains to identify barriers to SDF uptake and the policy actions required for effective implementation.

**Results:**

SDF is an effective, minimally invasive, and comparatively low‐cost intervention for arresting active carious lesions. Its simple application, limited equipment requirements, and suitability for community‐based delivery make it particularly relevant for children, older adults, people with special healthcare needs, and populations facing financial, geographic, or physical barriers to conventional dental care. However, broader implementation remains constrained by regulatory ambiguity, limited reimbursement and procurement pathways, workforce training gaps, aesthetic concerns, inconsistent consent and follow‐up processes, and fragmented governance. A six‐pillar framework is proposed, encompassing financing and procurement; regulatory clarity and professional scope; workforce training; patient communication, consent, and program monitoring; research and evaluation; and governance and intersectoral collaboration.

**Conclusions:**

The principal challenge to wider SDF integration is no longer the absence of supporting evidence but persistent policy and health‐system barriers. Incorporating SDF into oral health policy, alongside appropriate clinical protocols, referral pathways, financing, workforce preparation, and monitoring, could expand access to minimally invasive care and support more equitable, accessible, and sustainable oral health systems.

## 1 Introduction

Dental caries is the world's most common non‐communicable disease, disproportionately affecting children, older adults, and populations with limited access to conventional dental care. Despite advances in prevention, millions remain untreated because of financial, geographic, and physical barriers [1].

Silver diamine fluoride (SDF) is a simple, effective, and cost‐efficient topical agent for arresting active carious lesions, with particular value in minimally invasive and prevention‐oriented models of care. Its advantages include painless application, the absence of drilling or anesthesia, and suitability for delivery beyond traditional clinical settings. These characteristics align with key public health priorities, including minimally invasive care, cost containment, and improved access [2].

SDF is particularly relevant for children, older adults, and individuals with special health care needs. At the same time, its uptake may be limited by recognized disadvantages, including permanent black staining of treated lesions, occasional transient mucosal irritation, and esthetic concerns for patients and caregivers [3]. Despite these limitations, SDF has demonstrated important economic and service‐delivery advantages in the literature, with systematic reviews and economic evaluations suggesting favorable cost‐effectiveness and lower downstream treatment costs in appropriate settings [4, 5]. These advantages may be especially relevant for populations in whom conventional treatment is difficult, risky, or costly. In Australian children with special health care needs, for example, a single SDF intervention has been estimated at approximately AUD 190 and may help avert procedures under general anesthesia, which can be substantially more costly, thereby generating savings for families and the health system [6].

Recognizing these attributes, the World Health Organization (WHO) added SDF to the 2021 Model List of Essential Medicines, establishing an important global precedent for its integration into national health systems [7]. However, despite WHO endorsement and a substantial evidence base supporting its efficacy and safety, explicit policy uptake remains uneven and limited in many settings. This disconnect suggests that the principal challenge is no longer whether SDF works, but why health systems have been slow to translate evidence into regulation, financing, workforce integration, and routine care. Understanding this policy gap is essential to realizing the full equity potential of SDF.

### 1.1 The Policy Paradox: Evidence Without Implementation

The evidence base supporting SDF is substantial and well established. Systematic reviews, clinical trials, and professional guidance consistently support its safety and effectiveness across diverse populations and care settings [8, 9, 10], yet formal policy integration remains limited.

Several countries have developed partial or localized frameworks, including fluoride guidance in Australia, primary healthcare protocols in Brazil, and caries management recommendations in Japan [11]. However, these efforts remain fragmented, and no widely adopted clinical or policy standard currently ensures the consistent and equitable implementation of SDF at the population level [11].

This gap cannot be explained by scientific uncertainty alone. Rather, it reflects bureaucratic and institutional hesitation shaped by several interrelated factors. Regulatory ambiguity continues to slow progress, as approval pathways and product labeling have not always kept pace with the expanding clinical evidence base. Professional hesitation also remains important, particularly in relation to the black staining of arrested lesions and the esthetic concerns that may influence acceptance among clinicians, patients, and caregivers. In addition, rigid financing arrangements often exclude SDF from insurance reimbursement schedules and public procurement systems, further limiting access [12].

The transformation required is therefore not primarily scientific, but systemic [11]. Policy frameworks must evolve to clarify professional roles, support sustainable financing, and modernize workforce education in line with contemporary preventive and minimally invasive models of care. Without such reforms, health systems will continue to underuse a cost‐effective, evidence‐based intervention and, in doing so, reinforce inequities in access to oral healthcare, especially for populations facing the greatest barriers to conventional treatment [5, 6].

### 1.2 SDF as a Strategic Public Health Instrument

SDF should be understood not merely as a clinical adjunct, but as a strategic public health instrument capable of expanding access to care for populations underserved by conventional dentistry. Its significance lies in its simplicity of application, limited equipment requirements, scalability, and compatibility with preventive and minimally invasive care models. Its practical value also depends on reliable availability, coordinated procurement, and integration into existing public and community‐based service infrastructure [13]. Considered across three interrelated domains, clinical, workforce, and economic, SDF is particularly well positioned for integration into public oral health systems.

#### 1.2.1 Clinical Domain: Minimal Intervention and Patient Acceptability

The clinical value of SDF lies in its effectiveness in arresting carious lesions and in its capacity to reduce the procedural burden of care. By avoiding local anesthesia, rotary instrumentation, and complex restorative procedures, it offers a practical option for patients who may not tolerate invasive treatment or who face barriers to conventional dental services [14]. This is particularly relevant in pediatric, geriatric, and special‐needs settings, where referral for sedation or general anesthesia may increase cost, delay, and clinical risk. In young children with deeply carious lesions, SDF may also serve as an interim disease‐stabilizing option when immediate restorative treatment is not feasible, particularly where delay may increase pain, disease progression, or the likelihood of care under sedation or general anesthesia. This role requires careful case selection and clear referral pathways for lesions with pulpal or urgent care needs [15]. Such advantages are especially important for older adults and individuals with disabilities or special health care needs who may face transportation difficulties, dependence on caregivers, mobility limitations, or other logistical barriers to conventional clinic‐based treatment, making community‐level delivery particularly feasible and valuable [16, 17].

SDF should not be regarded as a direct substitute for restorative treatment in all circumstances. Unlike standard restorative approaches used in public dental services, including glass ionomer‐based restorations and atraumatic restorative treatment, SDF does not rebuild tooth form or function. Its principal role is to arrest active lesions and limit disease progression, particularly when conventional treatment is not feasible, acceptable, or immediately available. However, SDF may also be used as an adjunct to restorative care, including before or beneath glass ionomer‐based restorations in silver‐modified atraumatic restorative techniques, and in selected cases before preformed metal crown placement using the Hall Technique [4, 18, 19, 20].

Acceptability, however, is context dependent and extends beyond patient preference alone. Although the black staining of arrested lesions may limit uptake in some cases, many patients and caregivers consider this an acceptable trade‐off when weighed against pain, treatment avoidance, or limited access to conventional care [21]. Clinical acceptability may also vary according to tooth location, esthetic expectations, care setting, and the availability of restorative alternatives. In some settings, uptake may be further influenced by reimbursement structures or practice models that favor restorative intervention over non‐surgical management. These trade‐offs can be managed through careful case selection, clear informed consent, communication about expected outcomes, and referral pathways when esthetic or restorative priorities predominate [22].

From a public health perspective, these characteristics support the integration of SDF into community‐based anti‐caries programs across diverse settings, including preschool and kindergarten‐based early childhood caries prevention programs, school and special‐care school services, residential aged‐care facilities, rural and remote outreach programs, and primary care or community health settings where infrastructure, equipment, and specialist personnel may be limited. For example, in a preschool or community screening program, children with active cavitated lesions could receive SDF during the same visit, while those requiring restorative, pulpal, or urgent care could be referred through defined pathways. Evidence from school‐ and community‐based programs suggests that SDF can be incorporated into broader prevention strategies, not as a replacement for comprehensive dental care, but as a pragmatic disease‐control measure that can arrest active lesions, reduce treatment escalation, and create a bridge to further restorative or specialist care when required [2, 4, 23].

#### 1.2.2 Workforce Domain: Task Delegation and Workforce Flexibility

Compared with traditional restorative procedures, SDF application involves relatively low technique sensitivity and limited equipment requirements [2]. These characteristics make it well suited to delivery by appropriately trained non‐dentist providers, including dental hygienists, dental therapists, nurses, and community health workers, within clearly defined protocols and regulatory frameworks. The feasibility of such delegation, however, varies across jurisdictions and depends on local scope‐of‐practice rules, supervision requirements, regulatory approval pathways, acceptance within the dental profession, and clear referral pathways for cases requiring more complex care. Where these conditions are in place, policy mechanisms that support delegation can expand service capacity and improve access in underserved, rural, and remote settings [11]. This flexibility also creates opportunities for integration within school‐based, aged‐care, and primary care outreach services, where oral health needs may be high but dental workforce availability is limited. Incorporating SDF into undergraduate dental and oral health curricula, as well as continuing professional development for primary care and oral health practitioners, may further strengthen workforce flexibility and support longer‐term service sustainability [2, 24].

#### 1.2.3 Economic Domain: Economic Value and System‐Level Efficiency

The economic case for SDF adoption is increasingly persuasive [4, 5], but different forms of economic benefit should be distinguished. At the patient and provider level, SDF may reduce the need for more invasive and costly procedures [25]. At the payer and insurer level, retrospective analyses, including longitudinal data from the United States Medicaid program, suggest that policy support for SDF use may be associated with increased uptake and lower downstream treatment costs. For example, treatment delivered by Expanded Practice Dental Hygienists (EPDHs) was associated with measurable quarterly savings per patient, largely through reducing progression to more complex restorative care or treatment under general anesthesia [25, 26]. These findings suggest potential budgetary and service‐efficiency advantages, particularly in resource‐constrained settings. Avoiding preventable escalation of care may also help preserve specialist capacity and reduce pressure on hospital‐based services [6, 25]. However, broader claims regarding cost‐effectiveness, procurement efficiency, and long‐term system‐wide savings should be interpreted cautiously and may depend on local financing arrangements, procurement structures, workforce models, follow‐up requirements, and patterns of service use. Formal cost–benefit and budget impact analyses therefore remain important priorities for future policy evaluation [25].

### 1.3 Policy Directives and Call to Action

For SDF to move from an optional clinical intervention to a strategically integrated element of public oral health systems, policy, regulatory, and administrative actors must address the systemic barriers that continue to constrain its uptake. The feasibility of such implementation is supported by existing preschool‐, school‐, and community‐based oral health randomized controlled trials [27, 28, 29] that have incorporated SDF within broader prevention strategies, including the CariedAway school‐based program in the United States, as well as by its practical suitability for outreach, aged‐care, and special care settings where conventional clinic‐based treatment may be difficult to deliver [30].

Digital supports may further strengthen implementation by facilitating patient and caregiver education, school‐ and community‐based recall reminders, telehealth follow‐up, referral tracking, and program monitoring, particularly where follow‐up, reapplication, or linkage to further care is required. In preschool, school, and outreach programs, these tools may support continuity after initial SDF application by helping services monitor lesion arrest, identify the need for reapplication, and coordinate referral where required. Similar approaches may also benefit older adults, caregivers, and other populations who face barriers to regular clinic‐based attendance [16, 31].

### 1.4 Toward a Globally Adaptable SDF Protocol

Recent evidence syntheses, including the Cochrane review on topical SDF for preventing and managing dental caries, support its potential role in caries management while also highlighting variation in outcomes, settings, and certainty of evidence across dentitions. Therefore, a globally adaptable SDF application protocol should define a minimum set of clinical and operational steps while allowing adaptation to local regulations, workforce models, product availability, and resource constraints. Core elements should include caries‐risk and lesion assessment; screening for pulpal involvement, abscess, or acute infection; informed consent that explains expected black staining, benefits, limitations, and alternatives; tooth cleaning and relative isolation; protection of soft tissues; careful application using a microbrush or comparable applicator; post‐application instructions; documentation of treated teeth and lesion status; follow‐up to assess arrest; reapplication where indicated; and referral pathways for lesions requiring restorative, pulpal, urgent, or specialist care [32]. Future evidence‐synthesis and implementation research should compare existing clinical trials, professional guidelines, and manufacturer instructions to identify areas of agreement and variation and to support the development of harmonized, context‐sensitive protocols.

Building on the clinical, workforce, and economic considerations outlined above, the following six policy pillars offer a practical framework for national implementation, including the operational requirements needed for safe, acceptable, and scalable delivery (Table 1).

**TABLE 1:** Implementation‐oriented policy pillars for national integration of silver diamine fluoride.

Policy pillars	Rationale	Policy actions	Purpose
Financial architecture and procurement	Clinical and economic value will not translate into equitable access unless financing and procurement systems support routine availability	Introduce reimbursement pathways for SDF application, review, and reapplication where indicated. Include SDF in essential medicines, formularies, and procurement schedules for community, school‐based, aged‐care, and primary care services. Support coordinated purchasing and distribution to avoid stock disruption, especially in rural and low‐resource settings. Tie funding models to recall systems so follow‐up can occur after initial treatment.	To ensure equitable financing, reimbursement, and reliable supply
Regulatory delineation and professional scope	Regulatory ambiguity, jurisdictional variation, unclear scope‐of‐practice rules, and limited professional consensus can restrict implementation and discourage providers and institutions	Issue guidance defining indications, contraindications, consent requirements, documentation standards, and provider responsibilities. Specify which provider groups may apply SDF, under what training, supervision, or standing orders. Define referral pathways for teeth requiring restoration, pulpal assessment, urgent care, or specialist management after arrest or non‐response. Align protocols with local legal and professional frameworks.	To clarify indications, legal protections, provider authorization, and referral responsibilities
Workforce training and education	Sustainable implementation depends on workforce readiness across oral health, primary care, and community settings	Integrate SDF into undergraduate and continuing professional education. Train providers in case selection, lesion assessment, application technique, consent, record keeping, recall scheduling, reapplication decisions, and referral criteria. Include communication training on staining, esthetic concerns, treatment alternatives, and shared decision‐making with patients and caregivers.	To build workforce competence, flexibility, and implementation readiness across settings
Patient communication, consent, and program monitoring	Public trust, patient acceptability, and accountable implementation require consistent communication and routine oversight	Develop standardized patient information and consent materials that explain expected staining, benefits, limitations, alternatives, and follow‐up needs. Establish documentation templates that record tooth treated, lesion status, consent, treatment date, recall interval, reapplication decision, and referral outcome where relevant. Set context‐appropriate follow‐up and reapplication schedules within service protocols. Monitor uptake, lesion arrest, reapplication, referral, adverse events, documentation completeness, patient‐, caregiver‐, and clinician‐reported acceptability, escalation to further care, and program‐level oral health outcomes where feasible. Use digital reminders, education tools, telehealth follow‐up, and referral tracking where feasible to support recall, reapplication, and continuity of care. Implement routine audit and quality assurance processes.	To standardize communication, consent, follow‐up, documentation, and quality assurance
Research and evaluation integration	Policy uptake should be refined through local implementation data as well as clinical efficacy evidence	Support operational research and implementation studies across care settings. Evaluate cost‐effectiveness, feasibility, equity impact, patient and clinician acceptability, adherence to follow‐up protocols, and sustainability. Use findings to refine protocols, recall intervals, reapplication pathways, workforce training, and referral systems.	To create evidence feedback loops for implementation, acceptability, and system impact.
Governance and intersectoral collaboration	Effective scale‐up requires coordination across sectors, service platforms, and levels of government	Establish national or regional oversight mechanisms to coordinate implementation, reporting, and policy review. Build partnerships across health, education, social care, training institutions, payers, and community organizations. Assign accountability for procurement, workforce authorization, consent standards, monitoring indicators, and quality assurance. Support phased implementation through pilot programs in underserved public health settings, with evaluation before wider scale‐up. Incorporate disposal, waste management, and environmental sustainability considerations into procurement and implementation planning for large‐scale programs.	To strengthen coordination, leadership, and accountability for scale‐up

## 2 Equity, Ethics, and Implementation Imperatives

Beyond technical and administrative considerations, the integration of SDF has important implications for equity in access to care. Its simplicity, affordability, and adaptability make it particularly relevant for individuals and communities who may otherwise face barriers to treatment because of geography, disability, age, socioeconomic disadvantage, or disruption related to conflict and humanitarian crises [33]. Its equity potential may be further strengthened through collaboration across public oral health services, community programs, and private practitioners, particularly where service gaps persist and conventional models of care do not adequately reach priority populations.

In such partnerships, government agencies can support financing, procurement, workforce training, regulatory guidance, reimbursement mechanisms, and program‐level monitoring, while private practitioners can contribute to screening, SDF delivery where appropriate, referral, follow‐up, restorative care, and management of cases requiring more complex treatment. Shared protocols and referral pathways may help ensure that SDF delivered in community or public health settings is connected to ongoing care rather than functioning as an isolated intervention. Such collaboration may be especially valuable in rural and remote communities, Indigenous populations, aged‐care services, and other underserved settings, where flexible and community‐oriented delivery models can expand access to minimally invasive care.

In resource‐poor and geographically isolated communities, including Indigenous communities, SDF may also function as an interim triage and disease‐stabilization service when definitive restorative care is not immediately available [16, 34]. In such settings, SDF can help arrest suitable cavitated lesions, reduce the risk of further progression, and create time for referral or follow‐up within an organized care pathway. Its triage role should be clearly defined: teeth with signs of abscess, facial swelling, systemic infection, or irreversible pulpal involvement require urgent referral, while suitable lesions without signs of acute infection may be stabilized and monitored until further preventive or restorative care can be provided [35].

Conversely, failure to integrate SDF into policy and routine care may reinforce existing inequities in oral healthcare access, particularly where conventional restorative services remain financially, geographically, or operationally out of reach. In this context, SDF policy integration can be understood as part of a broader effort to reduce avoidable disparities in oral health [36].

## 3 Conclusion

The inclusion of SDF in the WHO Model List of Essential Medicines provides an important policy signal for governments and health systems considering its broader integration. The central challenge is no longer the absence of evidence, but the persistence of policy and system barriers that limit equitable implementation.

If supported by appropriate financing, regulation, and workforce preparation, SDF may help expand access to minimally invasive and cost‐conscious oral healthcare, particularly for populations underserved by conventional models of care. In this context, policy integration of SDF represents a practical step toward more equitable and prevention‐oriented oral health systems.

## Funding

The authors have nothing to report.

## Ethics Statement

The authors have nothing to report.

## Conflicts of Interest

The authors declare no conflicts of interest.

## Data Availability

The data that support the findings of this study are available on request from the corresponding author. The data are not publicly available due to privacy or ethical restrictions.
